# Multi-site Calciphylaxis With Pulmonary Calcification in a Young Haemodialysis Patient

**DOI:** 10.7759/cureus.98294

**Published:** 2025-12-02

**Authors:** Vismaya Pillai, Charles Hall

**Affiliations:** 1 Nephrology, Royal Free Hospital, London, GBR

**Keywords:** calcified lung metastasis, calciphylaxis, ckd-mbd, end-stage renal failure, haemodialysis non-adherence, metastatic pulmonary calcification, multi-site calcification, penile calciphylaxis, pulmonary calciphylaxis, sodium thiosulphate

## Abstract

Calciphylaxis is a rare and life-threatening complication of end-stage renal failure (ESRF), typically presenting with painful cutaneous necrosis but occasionally involving the visceral organs. Metastatic pulmonary calcification (MPC) results from calcium deposition within the alveolar walls due to severe derangements in mineral metabolism, whereas pulmonary calciphylaxis is even rarer and characterised by vascular calcification with thrombosis and tissue ischaemia. We describe a 34-year-old man with congenital horseshoe kidney and recurrent papillary urothelial carcinoma who developed ESRF secondary to obstructive uropathy and demonstrated profound haemodialysis (HD) non-adherence. He presented, following three weeks of missed dialysis, with lethargy, weakness, and severe hyperkalaemia (8.6 mmol/L). Within days, he developed painful violaceous plaques on the thighs and penile shaft, which rapidly progressed to necrosis, consistent with aggressive calciphylaxis. He subsequently developed breathlessness and cough; CT pulmonary angiography showed bilateral ground-glass opacities with septal thickening. Despite antibiotics, opacities persisted, and bone scintigraphy revealed increased tracer uptake in the lungs and subcutaneous tissues, supporting a diagnosis of MPC rather than pulmonary calciphylaxis. Management included intensified HD with low-calcium dialysate, intravenous sodium thiosulphate, non-calcium phosphate binders, analgesia, and anticoagulation for pulmonary embolism. Although oxygenation improved, cutaneous and penile lesions remained painful with poor healing. Given extensive calcification, malignancy, and dialysis dependence, he was referred to palliative care. This case highlights the diagnostic role of bone scintigraphy in distinguishing MPC from pulmonary calciphylaxis and underscores the importance of HD adherence and multidisciplinary management in preventing fatal outcomes.

## Introduction

Calciphylaxis is a rare but life-threatening complication of end-stage renal failure (ESRF), most commonly characterised by painful cutaneous lesions and associated with high mortality [1,2]. Although predominantly a dermatological and vascular condition, calcification may occasionally extend to the visceral organs. Metastatic pulmonary calcification (MPC) is a separate entity defined by calcium salt deposition within the alveolar walls and lung interstitium secondary to severe disturbances in calcium phosphate metabolism [3]. Pulmonary calciphylaxis is even rarer, shares overlapping radiological features with MPC, and requires histological confirmation for diagnosis [4].

We present a young patient with ESRF who developed cutaneous and penile calciphylaxis, alongside radiological findings suggestive of either MPC or pulmonary calciphylaxis, occurring in the context of profound haemodialysis (HD) non-adherence.

## Case presentation

A 34-year-old man with a congenital horseshoe kidney and recurrent low-grade papillary urothelial carcinoma of the bladder first presented in June 2021 with bilateral hydronephrosis. He underwent transurethral resection of bladder tumour (TURBT) with bilateral JJ stent insertion, resulting in improvement of serum creatinine from 1300 μmol/L to 650 μmol/L. Between 2021 and 2024, he experienced multiple tumour recurrences requiring repeat TURBT, but frequently failed to attend follow-up appointments.

In September 2023, he commenced unplanned HD via a tunnelled line for ESRF secondary to chronic obstructive uropathy. Dialysis adherence remained poor, including a documented four-month period of complete non-attendance.

In February 2025, after three weeks without HD, he presented with lethargy, weakness, frank haematuria, and severe hyperkalaemia (8.6 mmol/L). Despite several capacity assessments confirming full understanding of risk, he intermittently refused dialysis during admission.

**Figure 1:**
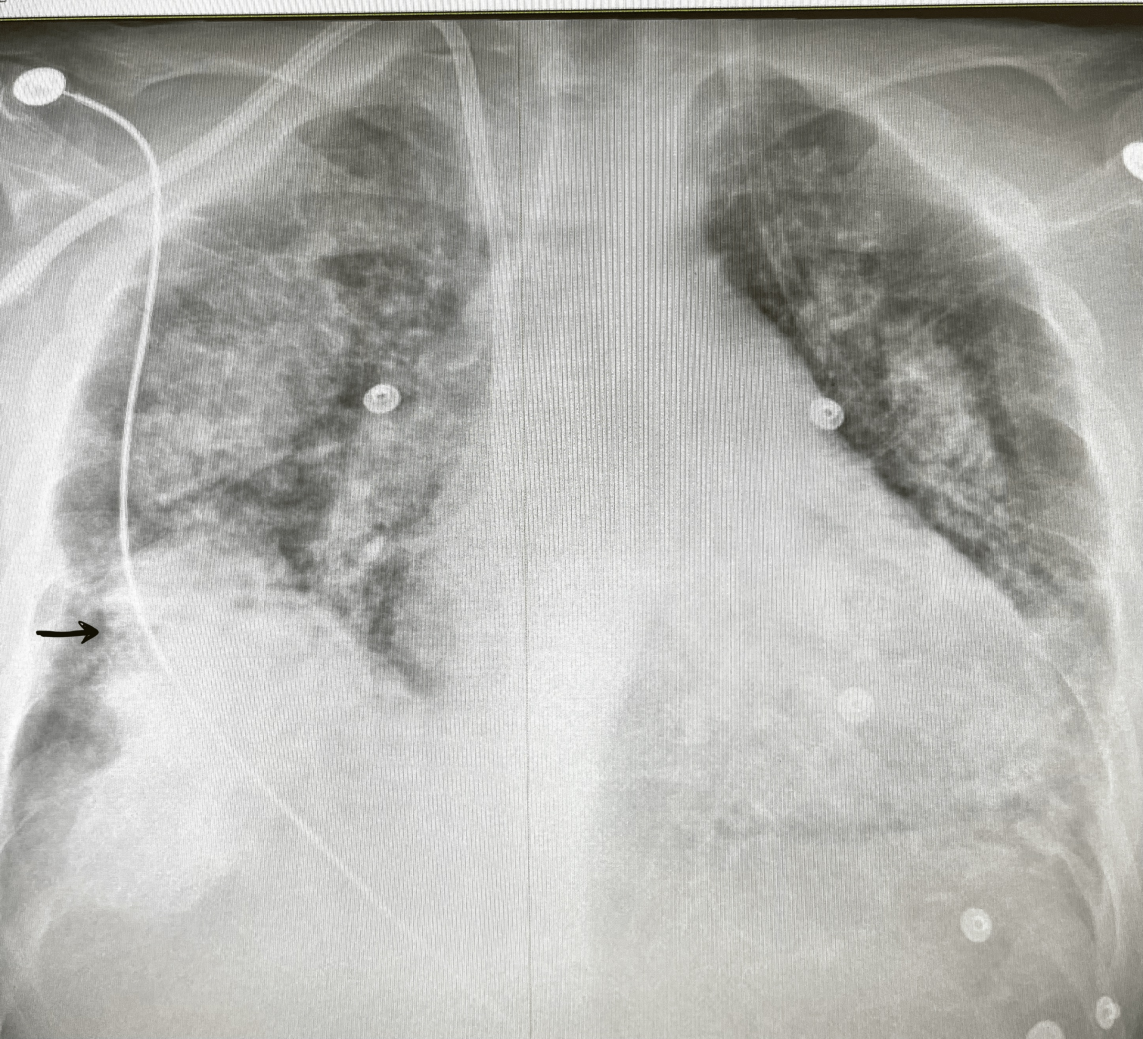
Chest radiograph showing bilateral patchy opacities This finding is non-specific and was initially treated as a possible infection, but later correlated with CT and bone scintigraphy, supporting a diagnosis of metastatic pulmonary calcification.

**Figure 2:**
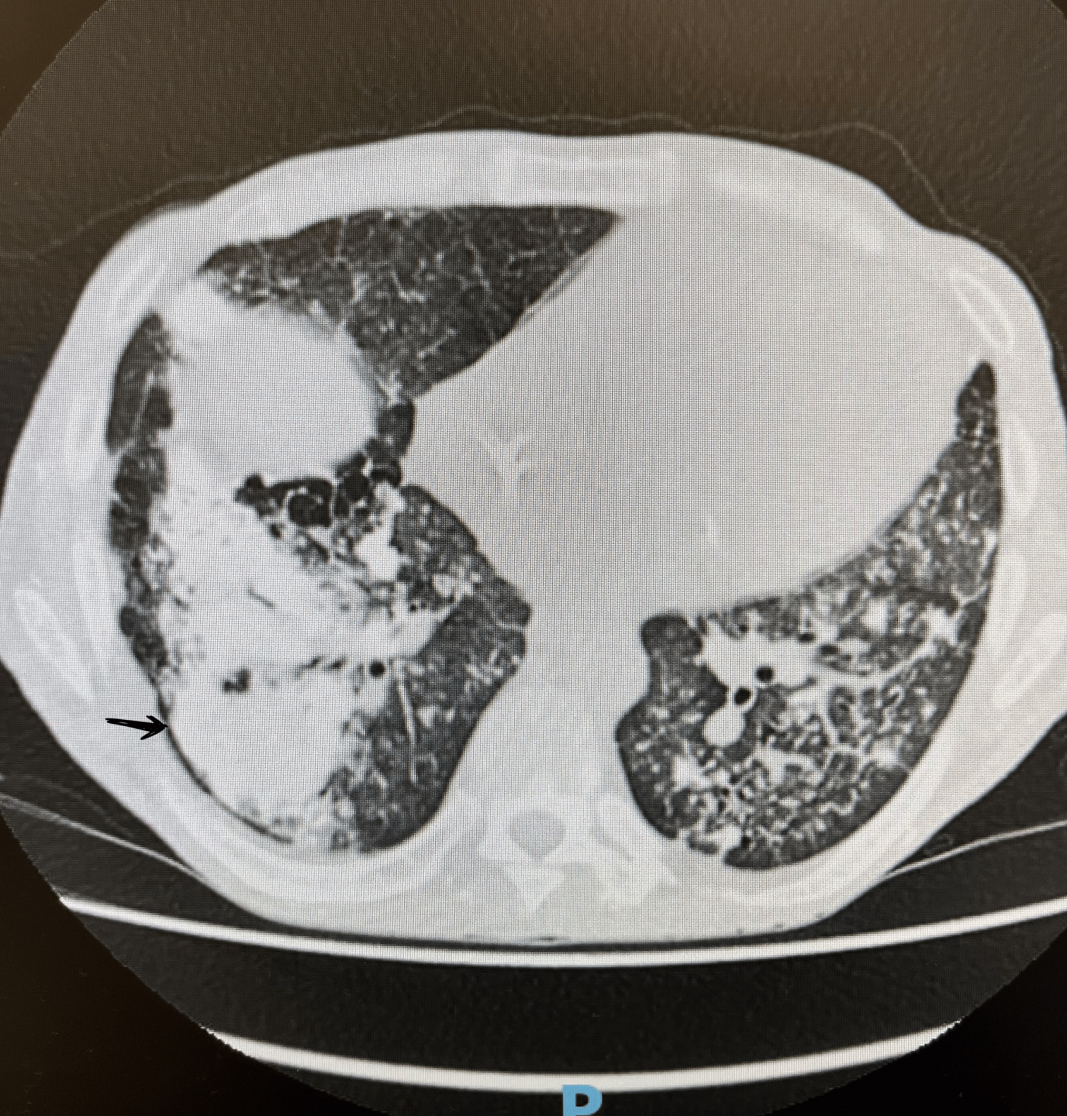
Chest CT demonstrating widespread bilateral ground-glass opacities with interlobular septal thickening This finding, in the context of end-stage renal failure and severe mineral imbalance, is consistent with metastatic pulmonary calcification.

**Figure 3:**
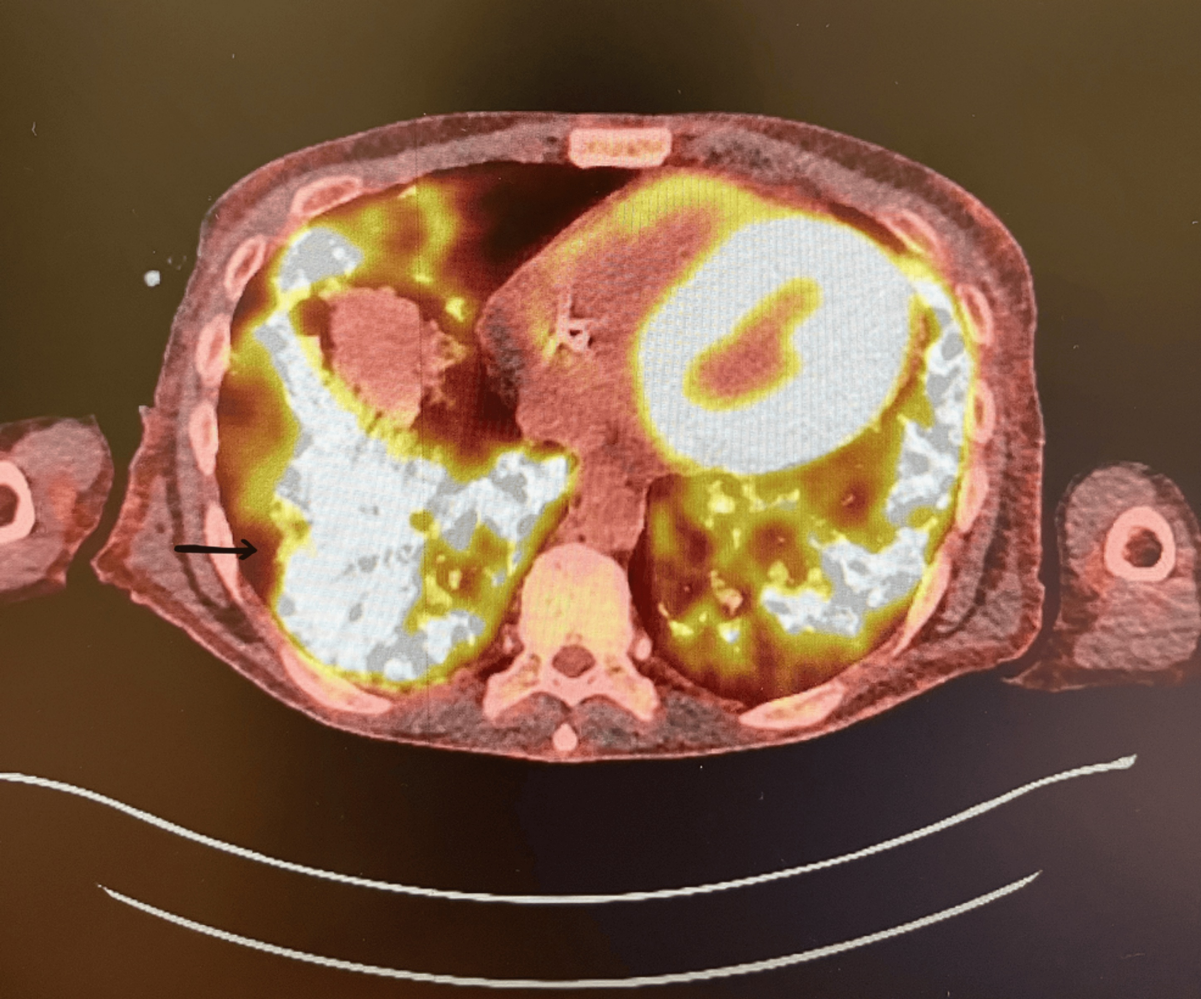
PET-CT of the chest demonstrating diffuse increased radiotracer uptake within bilateral lung fields, corresponding to ground-glass opacities This finding, in the setting of severe mineral imbalance, supports the diagnosis of metastatic pulmonary calcification rather than an infection or malignancy.

**Figure 4:**
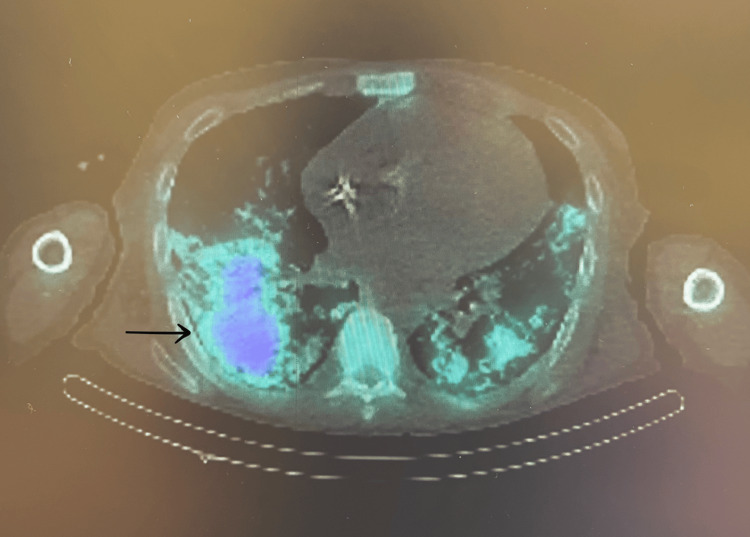
Bone scintigraphy with SPECT-CT showing increased tracer uptake in bilateral lung fields This finding, corresponding to ground-glass opacities on CT, is consistent with metastatic pulmonary calcification.

During this admission, he also reported progressive breathlessness, productive cough with brown sputum, fevers, and night sweats. Initial chest radiography showed non-specific bilateral opacities (Figure 1). CT pulmonary angiography demonstrated diffuse bilateral ground-glass opacities with interlobular septal thickening (Figure 2). Bronchoscopy was unremarkable, and both microbiological cultures and *Pneumocystis* PCR were negative. Persistent pulmonary changes despite antibiotics prompted nuclear medicine imaging. Bone scintigraphy demonstrated intense tracer uptake corresponding to the pulmonary opacities (Figures 3, 4), as well as uptake in the pancreas, sacroiliac joints, pubic tubercle, and left humerus (suggestive of a possible brown tumour) [3].

**Figure 5:**
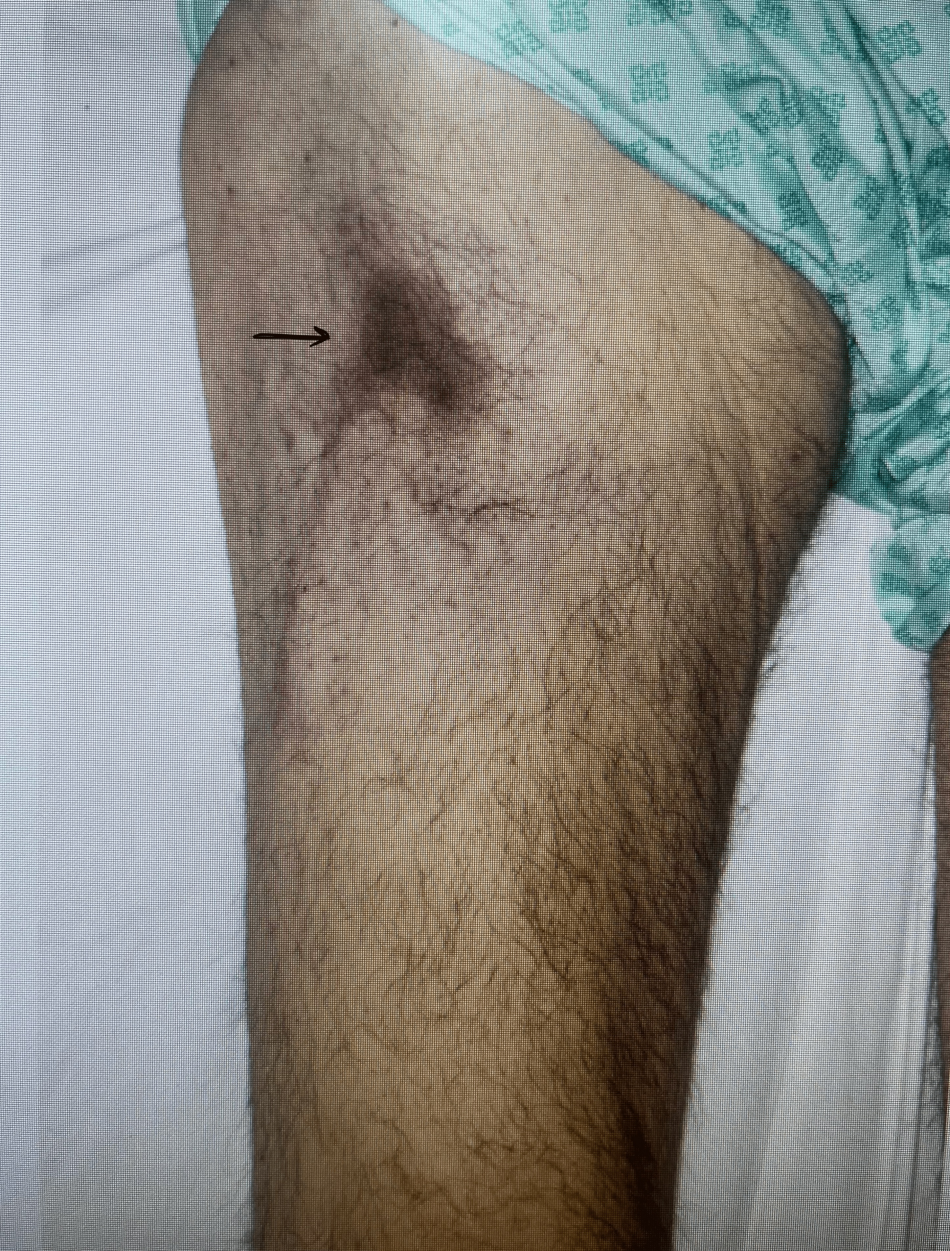
Early violaceous lesions over the anterior aspect of the thigh (June 2025)

**Figure 6:**
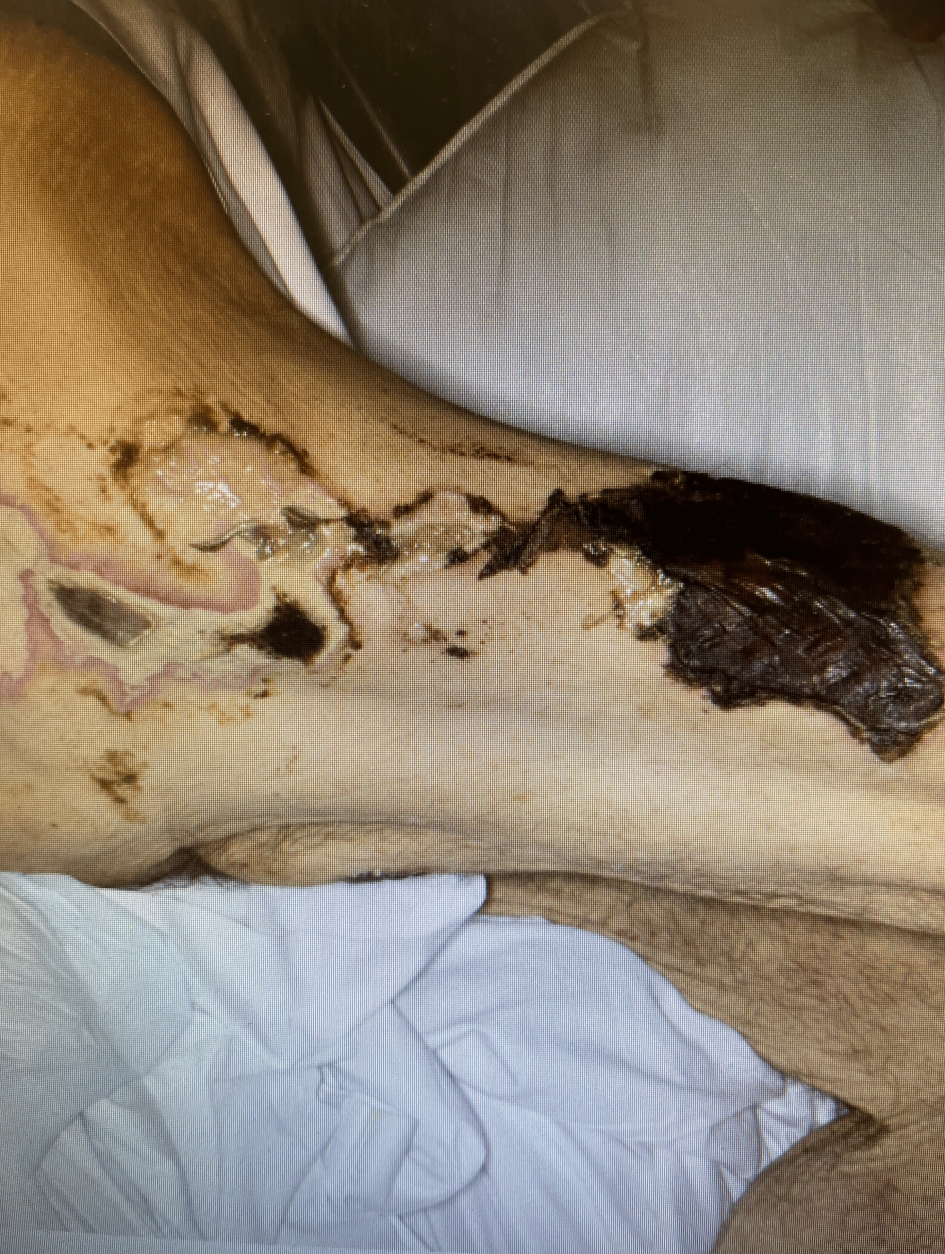
Lesion rapidly progressing to the anterior and lateral aspect of the thigh (August 2025) Within three months, the lesion rapidly progressed to the anterior and lateral aspect of the thigh, demonstrating induration and necrosis, with similar lesions on the penile shaft.

Soon afterwards, he developed rapidly progressive, exquisitely painful violaceous plaques over both thighs (Figures 5, 6), extending over days to indurated necrotic lesions. Similar changes developed on the penile shaft, consistent with calciphylaxis. Electromyography demonstrated severe proximal myopathy affecting the iliopsoas muscle; biopsy was not feasible.

Biochemistry demonstrated severe mineral dysregulation, including hyperphosphataemia, elevated calcium phosphate product, and secondary hyperparathyroidism.

Management included intensified HD with low-calcium dialysate, intravenous sodium thiosulphate (uptitrated to the maximum tolerated dose), non-calcium phosphate binders, broad-spectrum antibiotics for intercurrent infections, analgesia, and therapeutic anticoagulation following the diagnosis of pulmonary embolism.

Oxygen requirements improved prior to discharge; however, the cutaneous and penile lesions remained painful with limited healing. The patient remained dialysis-dependent with ongoing adherence difficulties. Given the extent of calcification across multiple organ sites, active bladder cancer, and poor overall prognosis, he was referred to palliative care with DNACPR in place and a ward-based ceiling of care.

## Discussion

This case illustrates an extensive multi-site calcification (cutaneous, penile, and pulmonary) in a young ESRF patient with profound HD non-adherence. Although calciphylaxis and MPC share similar biochemical drivers, such as hyperphosphataemia and secondary hyperparathyroidism [2], they represent distinct pathological processes. Calciphylaxis involves calcification and thrombosis of small vessels, resulting in tissue ischaemia and necrosis [1], while MPC is characterised by non-thrombotic calcium deposition in normal lung parenchyma due to chronic disturbances in mineral metabolism [3].

Pulmonary calciphylaxis was initially considered given the presence of widespread cutaneous disease. However, MPC was ultimately favoured because the pattern of uptake on bone scintigraphy aligned with reported features of MPC, and there was no histological confirmation to support pulmonary calciphylaxis [4]. Distinguishing between these entities is clinically important: MPC may stabilise or partially improve with correction of mineral imbalance, whereas pulmonary calciphylaxis is associated with rapid deterioration and markedly poorer outcomes [4].

Penile calciphylaxis, although uncommon, is recognised as a severe manifestation associated with significant pain, risk of secondary infection, and delayed healing [2]. In this patient, the rapid evolution from violaceous plaques to necrosis reflected an aggressive trajectory strongly influenced by prolonged periods without dialysis, the most significant modifiable contributor in his case. No clinical photographs of the penile lesions were obtained, as the patient declined consent for imaging. Prolonged HD non-adherence likely exacerbated hyperphosphataemia, elevated calcium phosphate product, and secondary hyperparathyroidism, all of which accelerate tissue calcification.

Despite intensified dialysis, low-calcium dialysate, sodium thiosulphate, and medical optimisation, the prognosis remained poor given the extent and speed of calcification. This case highlights the critical role of maintaining dialysis adherence and biochemical control in preventing severe systemic calcification and underscores the challenges of managing advanced disease once it becomes rapidly progressive.

## Conclusions

Calciphylaxis may involve unusual and functionally sensitive sites, including the penis, and can progress rapidly with severe morbidity. In ESRF patients presenting with calciphylaxis and unexplained pulmonary opacities, both pulmonary calciphylaxis and MPC should be considered, with bone scintigraphy serving as a valuable non-invasive diagnostic adjunct. Effective management requires aggressive biochemical optimisation, intensified dialysis, and coordinated multidisciplinary care. However, prognosis remains poor in extensive or rapidly progressive disease. This case also highlights the critical impact of dialysis adherence, as inadequate treatment and uncontrolled mineral imbalance significantly accelerate calcification and worsen outcomes.
